# Nutrition in Patients with Type 2 Diabetes: Present Knowledge and Remaining Challenges

**DOI:** 10.3390/nu13082748

**Published:** 2021-08-10

**Authors:** Maria Letizia Petroni, Lucia Brodosi, Francesca Marchignoli, Anna Simona Sasdelli, Paolo Caraceni, Giulio Marchesini, Federico Ravaioli

**Affiliations:** 1 IRCCS-Azienda Ospedaliera di Bologna Sant’Orsola-Malpighi, I-40138 Bologna, Italy; marialetizia.petroni@unibo.it (M.L.P.); lucia.brodosi2@unibo.it (L.B.); francesca.marchignoli@gmail.com (F.M.); annasimona.sasdelli@aosp.bo.it (A.S.S.); paolo.caraceni@unibo.it (P.C.); f.ravaioli@unibo.it (F.R.); 2 Department of Medical and Surgical Sciences, Alma Mater University of Bologna, I-40138 Bologna, Italy

**Keywords:** behaviour, diet, lifestyle, nutrition supplements, sarcopenia, type 2 diabetes

## Abstract

Unhealthy behaviours, including diet and physical activity, coupled with genetic predisposition, drive type 2 diabetes (T2D) occurrence and severity; the present review aims to summarise the most recent nutritional approaches in T2D, outlining unmet needs. Guidelines consistently suggest reducing energy intake to counteract the obesity epidemic, frequently resulting in sarcopenic obesity, a condition associated with poorer metabolic control and cardiovascular disease. Various dietary approaches have been proposed with largely similar results, with a preference for the Mediterranean diet and the best practice being the diet that patients feel confident of maintaining in the long term based on individual preferences. Patient adherence is indeed the pivotal factor for weight loss and long-term maintenance, requiring intensive lifestyle intervention. The consumption of nutritional supplements continues to increase even if international societies do not support their systematic use. Inositols and vitamin D supplementation, as well as micronutrients (zinc, chromium, magnesium) and pre/probiotics, result in modest improvement in insulin sensitivity, but their use is not systematically suggested. To reach the desired goals, patients should be actively involved in the collaborative development of a personalised meal plan associated with habitual physical activity, aiming at normal body weight and metabolic control.

## 1. Introduction

Diabetes mellitus, namely type 2 diabetes (T2D), constitutes a significant challenge for health systems worldwide. According to the 2019 Diabetes Atlas of the International Diabetes Federation [1], 463 million adults are currently living with diabetes (1 on 11 individuals worldwide, but 1 in 5 are aged over 65). The total number is expected to increase further by 700 million in 2045. The economic impact is huge—driven by the direct costs of treatment and complications, the indirect costs of disability and premature death, and the intangible costs of poor quality of life.

Despite its characterizations as a disease of affluence, nutritional problems are frequent in T2D. Unhealthy lifestyles expressed by overnutrition and/or scarce physical activity, leading to overweight and obesity, add to genetic defects in the pathogenesis of the disease. Dietary restrictions are prescribed to reduce the incidence of T2D as well as to improve metabolic control [2], but weight loss is burdened by the loss of muscle mass [3] and sarcopenia adds to age-dependent muscle wasting [4], increasing frailty [5]. These two opposite needs make a correct nutritional approach mandatory to reduce disease burden, improve metabolic control, limit pharmacologic treatment and reduce the risk of impending cardiovascular disease.

National and international guidelines for nutritional and lifestyle recommendations are available [5,6,7,8,9], together with protocols to guide weight loss to produce long-term T2D remission [10]. The proposed strategies (dietary prescription, lifestyle counselling, cognitive behaviour therapy), although all-inclusive of nutritional components, are markedly different in their approach and goals and should be known by clinicians approaching patients with T2D (Table 1) [11]. The present review is intended to summarize the most recent nutritional approaches in T2D, also outlining unmet needs.

## 2. Methods and Areas of Research

### 2.1. Literature Search

The literature on T2D is immense. A PubMed search of June 2021, limited to the period 2016–2021 using the string “Type 2 diabetes” [MeSH Terms] AND “nutrition” [All Fields] AND “human” [MeSH Terms], retrieved 4865 references, including 887 review articles (234 systematic reviews), 255 meta-analyses and 760 clinical trials. The authors used the search to enucleate the most relevant data and unmet treatment needs. The reference lists of selected articles were used to retrieve older documents in order to provide a complete overview of present problems.

### 2.2. Diabetes, Obesity and Sarcopenia

The association between T2D and obesity is so strict that the term “diabesity” was originally used to indicate the dreadful association of the two conditions in a JAMA editorial in 1980 [12]. The term was finally proposed by Astrup and Finer [13], as well as by Zimmet et al. [14] and it is largely accepted inside the metabolic community. The accumulation of body fat characterizes obesity, but it is measured by a formula (the body mass index, i.e., weight (kg)/height^2^ (m)), not at all considering body fat. Muscle mass is frequently increased in obesity but might be relatively scarce in quantity and quality compared to body fat.

Sarcopenia is particularly common in older patients, synergistically driven by age and obesity; body fat increases until the seventh decade of life (the median age of patients with diabetes attending diabetes centres) and decreases thereafter [15]. At the same time, sedentariness progressively reduces muscle mass, finally resulting in sarcopenic obesity [16], frequently associated with cardiometabolic disorders [17].

By definition, sarcopenia implies a quantitatively reduced muscle mass, as measured by dual-energy X-ray absorptiometry (DXA), the commonly accepted gold standard. Several studies have validated the use of bioelectrical impedance analysis (BIA), an easy, time-saving, and cost-effective bedside technique for assessing regional muscle mass and body composition [18,19]. BIA-assessed sarcopenia is defined by the skeletal muscle mass index (SMI), calculated as total appendicular skeletal mass (ASM, kg) divided by body weight (kg) × 100. These measurements do not consider qualitative muscle mass, and most recent guidelines suggest that functional measurements (e.g., low muscle strength by handgrip) should be primarily used to characterize sarcopenia, with quantitative data as supportive measures [20].

The prevalence of sarcopenia in diabetes has been extensively investigated. In a recent narrative review, the prevalence of sarcopenia varied between 7% and 29% [21], according to age and metabolic control, but higher figures are frequently reported. A systematic review with meta-analysis including 15 studies confirmed a prevalence varying up to 50% [22], again driven by age and metabolic control. A study with BIA concluded that patients with T2D have an enlarged ectopic fat at the expense of skeletal muscle, i.e., relative sarcopenia [23], and lower muscle mass is coupled with decreased muscle strength [24], also predicting diabetes in the general population [25]. The contribution of diabetes duration remains controversial [21,22], but older patients with T2D, with an expected longer duration of disease, show a larger decline in appendicular lean mass, muscle strength, and functional capacity compared with normoglycemic controls [26]. Notably, when compared with matched control populations, the risk of sarcopenia increased systematically in the presence of T2D (odds ratio (OR) 1.55; 95% confidence interval (CI) 1.25–1.91; *p* < 0.001 [22] and OR 1.63; 95% CI 1.20–2.22; *p* = 0.002 [27]). This indicates a need for preventive measures to limit quantitative and qualitative muscle defects by effective nutritional treatments.

### 2.3. Metabolic Control

The primary defect in T2D is insulin resistance, a condition where normal insulin levels are associated with lower metabolic effects or where higher than normal insulin levels are needed to elicit a normal metabolic response. Insulin resistance accounts for diffuse impairment in whole body, as well as in selective defects in different organs and tissues (liver, muscle, adipose tissue).

Whole-body insulin resistance mainly reflects muscle insulin resistance [28], reducing glucose and amino acid uptake in the postprandial phase, as well accelerating glycogen and amino acid release in the post-absorptive state, also accelerated by glucagon release [29]. Glucagon constitutes the link between muscle and liver in substrate disposal; by stimulating hepatic glucose production and ketogenesis, glucagon favours the utilization of substrates released in the periphery, whereas high insulin concentrations favour hepatic fat deposition. In both obese and nonobese subjects, higher plasma insulin levels have been associated with a linear increase in the rates of hepatic de novo lipogenesis [30], as supported by the hypoglycaemic effects of glucagon suppression of glucagon-receptor antagonists [31,32]. In the hepatocytes, fatty acids may be derived from de novo lipogenesis, uptake of non-esterified fatty acids and low-density lipoproteins, or lipolysis of intracellular triacylglycerol. Their accumulation due to higher synthesis and decreased export in the presence of high insulin concentrations in the portal vein is the likely cause of fatty liver disease, occurring in up to 73% of patients with T2D [33].

**Figure 1:**
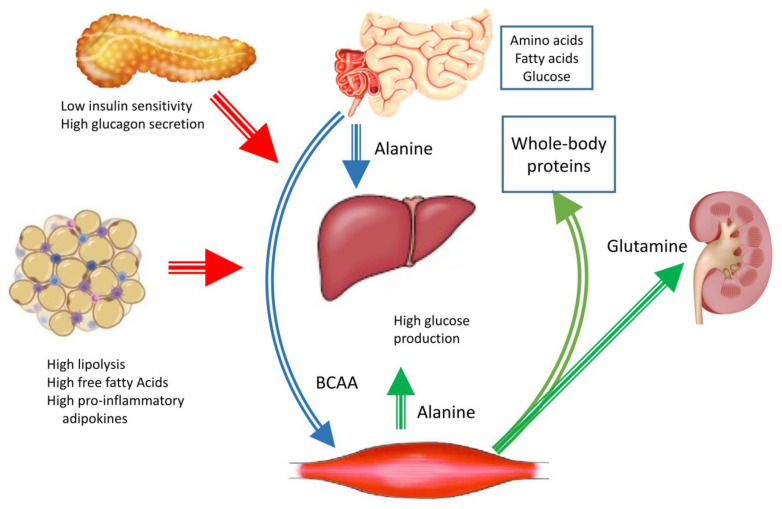
Interorgan amino acid exchange in the postabsorptive state and after meals in diabetes. Note the importance of BCAAs (valine, isoleucine and leucine) as nitrogen carriers to the muscle tissue (lean mass) in the post-prandial period (blue arrows) and the reverse importance of alanine and glutamine as nitrogen carriers to central organs in the post-absorptive state (liver, kidney, intestine) (green arrows). In this context, the regulatory role of the pancreas (altered secretion of insulin and glucagon) and the adipose tissue (lipolysis, release of free fatty acids and inflammatory adipokines in the general circulation, particularly in the post-absorptive state) is pivotal for the regulation of hepatic and whole-body homeostasis (red arrows).

The link between muscle tissue and the liver is exerted by amino acids (Figure 1) [34]. Branched-chain amino acids, bypassing the liver in the post-prandial state, serve as nitrogen carriers to the periphery, whereas alanine and glutamine are used to carry nitrogen from the periphery to the liver, intestine and kidney. In insulin-resistant states, including obesity [35], the post-load uptake of branched-chain amino acids is impaired, possibly leading to defective amino acid supply to the muscle tissue and sarcopenia. In summary, the complex trafficking of glucose, lipid and amino acid in response to insulin resistance should be considered in the treatment of diabetes.

## 3. Medical Nutrition Therapy for Type 2 Diabetes

The foundation of medical nutrition therapy (MNT) of T2D is to achieve glucose, lipids, and blood pressure within the target range to prevent, delay or manage microvascular and macrovascular complications [36,37].

MNT plays a pivotal role in the overall management of diabetes, and patients with T2D should be actively involved with their healthcare team for the collaborative development of a personalized meal plan. If these patients are referred to a registered dietitian or a nutritionist proficient in providing diabetes-specific treatment, an absolute reduction of glycated A1C haemoglobin of up to 1.9% may be observed [8]. Continuous dietary counselling integrated with mobile apps and wearable devices has also been advocated to facilitate the real-time assessment of dietary intake, to strengthen adherence, and support motivation and self-efficacy [38].

### 3.1. Comparison between Different Guidelines

Table 2 summarizes the main nutritional recommendations for patients with T2D, derived from guidelines, and the dietary patterns with a high degree of evidence [5,6,7,8,9]. All proposed interventions are designed to reduce energy intake and promote 5–10% loss of initial body weight, leading to improved insulin sensitivity, blood glucose and blood pressure control, and reduced lipid levels [39]. Regular mealtimes and a healthy diet should be combined with increased physical activity [4].

The optimal distribution of macronutrients as a percentage of total energy is highly variable, from 45% to 60% for carbohydrates, from 15% to 20% for proteins and 20% to 35% for fats, suggesting no ideal percentage of calories from macronutrients [7]. As to carbohydrates, high-fibre sources (30–50 g/day of dietary fibre, ≥30% as soluble fibres) and minimally processed, low-glycaemic index carbohydrates should be preferred to improve glycaemic control, LDL-cholesterol and cardiovascular (CV) risk. Overall, reducing carbohydrate intake for individuals with T2D has been shown to improve blood glucose [6]; a systematic review and meta-analysis (9 studies with 734 patients) confirmed a beneficial effect of low-carb diets vs. normal-or high-carb diets on HbA1c and on short-term weight loss, not on long-term weight loss [40]. Food plans should emphasize the consumption of non-starchy vegetables, with minimal added sugars, fruits, whole grains, and dairy products [41]. Using non-nutritive sweeteners as substitutes for added sugar (sucrose, high fructose corn syrup, fructose, glucose) can reduce daily calories and total carbohydrates. For those who regularly consume sugary drinks, consuming a low calorie or unsweetened drink can be an alternative, but both should be consumed with caution.

Additionally, recommendations on protein intake do not differ from the general population (1.0–1.2 g/kg body weight or corrected body weight for patients with overweight/obese); protein intake should be reduced to 0.8 g/kg body weight in subjects with chronic diabetic nephropathy [36]. At present, there is some inconsistency across guidelines from different countries as to protein sources (some do not limit animal proteins) and as to allowed maximal amount of protein intake (1.2–1.5 g/kg/day) [42]. A recent meta-analysis of 54 RCTs (4344 participants) showed a significant effect of moderate high-protein diets (20–45% of total energy) vs. low-protein diets (10–23%) on weight loss and weight loss maintenance, total fat mass reduction and cardiometabolic risk [43]. The authors suggest that the effects might also be due to the blood-pressure-lowering effect of bioactive peptides that inhibit the angiotensin-converting enzyme activity observed in protein isolates [44].

Among dietary fats, it is recommended to avoid trans-fatty acids as much as possible and to consume less than 7–9% of the total daily energy from saturated fatty acids (SFAs). SFAs should be replaced with polyunsaturated fatty acids (PUFAs), mainly mixed sources of omega-3/omega-6, and with monounsaturated fatty acids (MUFAs) of vegetable origin whole grains, nuts and seed (rich in alpha-linolenic fatty acid) [36,45].

The recommendations have largely focused on the quality of the diet and the importance of a healthy eating pattern that contains nutrient-rich foods, with less attention to the percentage of specific nutrients, with a reduction in daily caloric intake (250–500 kcal) for subjects with overweight and obesity [6]. Several dietary patterns have been studied and proposed, but no single dietary pattern should be preferred [8]. Individual preferences and treatment goals will determine the long-term use of these models; systematic reviews and meta-analyses have shown that a Mediterranean-style dietary pattern significantly improves hard outcomes such as glycaemic control, systolic blood pressure, total cholesterol, HDL-cholesterol and triglycerides [46]. The Mediterranean diet is characterised by a moderate-to-low carbohydrate intake, entirely covering the micronutrient needs [47]. Additionally, a low fat diet, i.e., the DASH-diet, promoted in the prevention of cardiovascular disease and the treatment of high blood pressure [48], has also reached consensus [49]. In a review comparing low-carbohydrate and ketogenic diets, the vegan diet, and the Mediterranean diet, all diets improved glycaemic control and weight loss, but patient adherence and long-term manageability were pivotal factors for the efficacy of each diet [50].

### 3.2. Intensive Lifestyle Intervention

Intensive lifestyle intervention (ILI) that supports behaviour changes, as initially experienced in the Finnish Diabetes Prevention Study and the U.S. Diabetes Prevention Program [51,52], represents the recommended approach to prevent and/or delay the onset of T2D in prediabetic patients [5]. The ILI behaviour approach combines diet and physical activity interventions with the goal to achieve and maintain a 7% loss of initial body weight and to increase moderate-intensity physical activity to at least 150 min/week. The effect of ILI has also been investigated in the treatment of T2D. The Look AHEAD study randomized 5145 individuals with T2D and associated overweight or obesity to either ILI or diabetes support and education (as control group), having cardiovascular outcomes as primary goal. Weight loss was achieved by reducing caloric intake to 1200–1800 kcal/day depending on baseline weight using portion-controlled meal plans, calorie-counting techniques, and meal replacements combined to moderate physical activity to ≥175 min/week. ILI was delivered as individual and group sessions over the first year, with a median follow-up of 9.6 years. [53]. After one year, the average weight loss in the ILI group was 8.6%, compared with 0.7% in the control group, with 55% of ILI participants having lost ≥7% of their initial b.w. vs. 7% of controls. This led to remission of T2D in 11.2% of ILI participants vs. 2.0% in controls. However, by the fifth year of follow-up, ILI participants had regained half of their initial weight loss, and the study was closed at the end of the follow-up (10-years) after an interim analysis had shown that the intervention had failed its primary outcome [54]. Thus, the critical point becomes how to achieve long-term weight loss maintenance, a difficult task in the general population [55], and a core problem in T2D treatment with approaches based on lifestyle changes. Although more effective than behaviour change in inducing and sustaining remission of T2D, bariatric surgery also suffers from reduced durability over time [56].

A novel approach was tested in the DIRECT trial, a primary care-led management intervention in patients with T2D diagnosed by less than 6 years and not receiving insulin. The ILI strategy was preceded by a commercial very-low-calorie diet followed by stepwise food reintroduction. Primary outcomes were weight loss ≥15 kg and T2D remission. At 12 months, almost half of participants achieved T2D remission off all glucose-lowering medications [57]; this percentage dropped to 36% at 24 months [58]. Notably, the maintenance of diabetes remission paralleled weight loss maintenance and particularly fat removal from the liver and pancreas, suggesting recovered insulin secretion [59]. With the limits of durability, all these data support the use of ILI, including dietary interventions, as an effective adjuvant treatment to improve glycaemic control [60].

Another approach is the so-called intermittent fasting, which has gained increased popularity for treating T2D based on very limited literature [61]. This term encompasses various eating behaviours that avoid (or limit) nutrient and energy intake for a significant amount of time (a full day or a time-restricted feeding between 6 to 8 h) on a regular intermittent schedule. Intermittent fasting is claimed to improve glucose control, insulin resistance and to induce weight loss by generating a ‘metabolic switch’, i.e., a sort of rejuvenation of the metabolic homeostasis, leading to increased health span and longevity [62], but no advantage over conventional caloric restriction has been proven. Moreover, this regimen could carry the risk of hypoglycaemia even when following a medication dose-change protocol and should only be used under strict medical control and/or continuous glucose monitoring [63].

Finally, the use of mobile apps and wearable devices has recently gained consensus to facilitate weight loss. The use of these devices allows a direct analysis of daily calorie intake and physical activity (daily steps), translated into calorie consumption [64]. This provides immediate feedback and is likely to support long-term adherence to well-defined goals [38]. Several commercial apps are available, and have been tested in the prevention and treatment of diabetes in trials mimicking the U.S. Diabetes Prevention trial [52]. Toro-Ramos et al. confirmed a modest efficacy of weight loss for app users after 6 and 12 months of systematic use in subjects with prediabetes compared with usual care [65], and similar studies are available with the most recent apps that also support by tailored messages interactivity [66]. Although all these supports are expected to improve long-term weight loss, and a few patients may really reach impressive results [67], their use is biased by higher attrition rates [68]. Nonetheless, the possibility to reach a larger audience makes this approach a useful opportunity.

## 4. Nutritional Supplements for Metabolic Control

International diabetes societies do not support the use of nutritional supplements in diabetes, but their use continues to increase in several countries, despite lack of evidence and uncertainty on safety [36]. A complete analysis of available products (combinations may account for several hundreds) is outside the scope of this review, but a few of them are of interest. Their putative mechanism(s) of action are summarized in Table 3 [69,70,71,72,73,74,75,76,77,78,79,80,81,82,83,84,85,86,87,88,89]. They are not expected to replace diet and glucose-lowering drugs but might be confidently used, provided their safety is proven.

### 4.1. Inositols

Several reviews and meta-analyses have been published on the treatment of gestational diabetes with myo-inositol (MI) or D-chiro-inositol (DCI) [70,90,91,92,93]. A Cochrane review was inconclusive [94]; MI supplementation did not reduce the need for insulin or produce any significant effect on blood glucose. Conflicting data have also been reported using DCI or the combination of MI and DCI, and the optimum dosage to achieve a significant effect on glucose metabolism remains unsettled [91]. A position statement of the two largest Italian diabetes societies concluded that MI (at the dose of 4 g/day) might be safely used for the prevention and treatment of gestational diabetes [95], but the level of evidence and the strength of recommendations are low. No data are available on the use of MI or DCI to treat insulin resistance outside gestational diabetes. Studies are in progress on the combined use of MI and myo-inositol hexa-phosphate (IP6), or phytic acid, showing more effective anti-oxidant and glucose-lowering activity in experimental animals [96], but no clinical data are available.

The use of inositol(s) in polycystic ovary syndrome is not considered in the present review; in that setting, specific hormonal activity is likely to produce clinical effects [97].

### 4.2. Vitamin D

Vitamin D levels are frequently suboptimal in T2D, probably driven by overweight/obesity, and specifically by visceral adiposity [98], and have been associated with chronic inflammation and insulin resistance, as well as impaired insulin release [99]. Epidemiological studies support the existence of a relationship between low vitamin D levels and the presence of T2D, metabolic syndrome [100,101], nonalcoholic fatty liver disease (NAFLD) [102], cardiovascular risk factors [103] and insulin resistance, also tested by glucose clamp [75]. However, a clear association between vitamin D levels, insulin and glucose metabolism has not been systematically confirmed by intervention studies, and a causal association has never been established [104]. In a subset of the RECORD trial, a placebo-controlled trial of oral vitamin D_3_ and/or calcium supplementation for the secondary prevention of osteoporotic fractures in older people, vitamin D_3_ at the daily dose of 800 IU with or without 1000 mg of calcium did not prevent the development of T2D and did not reduce the need for glucose-lowering drugs in T2D patients [105]. Although the effects on insulin sensitivity have long been conflicting [73], a recent systematic review with metanalysis confirmed that vitamin D supplementation resulted in a significant improvement in HOMA-IR (standardized mean difference = −0.57; 95% CI: −1.09 to −0.04), particularly when vitamin D was administered in large doses and for a short period of time to nonobese, vitamin D deficient patients, or to individuals with optimal glucose control at baseline [106]. Data have been confirmed in another recent study in vitamin D-deficient adults randomized to high dose vitamin D supplementation. The HOMA value of insulin resistance was significantly reduced, and a lower rate of progression toward diabetes was observed vs. the control group (3% vs. 22%; p = 0.002) [107].

Of note, vitamin D has been extensively used also to treat sarcopenia, considering the role of insulin resistance extending from glucose metabolism to protein and amino acid metabolism, as discussed below.

### 4.3. Niacin

Niacin is a water-soluble derivative of pyridine, present in several forms (namely as nicotinic acid or nicotinamide), also named as vitamin B3. It is a derivative of vitamin B, frequently associated with inositols as inositol hexanicotinate. The effects on insulin release from islet β-cells have been extensively investigated in T2D with secondary failure of sulfonylureas, where niacin at the daily dose of 1.5 g significantly restored C-peptide release [108]. However, a meta-analysis of eight trials where niacin was used to treat hyperlipidemia in 2110 T2D patients showed no significant effects on plasma glucose (weighted mean difference (WMD), 0.18 mmol/L; 95% CI, −0.14 to 0.50) and HbA1c levels (WMD, 0.39%; 95% CI, −0.15 to 0.94) [109]. Niacin appeared to cause a deterioration of glucose control, in keeping with data observed in a meta-analysis of 11 trials in patients without diabetes at entry, where niacin was used to treat dyslipidaemia and prevent cardiovascular events [110] (relative risk of de novo T2D: 1.34 (95% CI 1.21–1.49)). Similar results were provided by the large trial of combination treatment with niacin plus laropiprant [111], where niacin treatment (2 g/day for a median of 3.9 years) was associated with an increased incidence of de novo T2D (rate ratio, 1.32; 95% CI 1.16–1.51) and deterioration in metabolic control in subjects with diabetes (1.55; 1.34–1.78) [112]. This deleterious effect is similar to the well-known, mild negative effect of statins on glucose metabolism. It adds to the well-known poor tolerability of niacin because of flushing, occurring at pharmacologic doses.

### 4.4. Nutraceuticals

Natural compounds derived from plant extracts, spices, herbs, and essential oils have been tested for alleged benefits in managing patients with metabolic syndrome [77,113]. They include Mediterranean diet components, olive oil and its anti-oxidant components, natural legumes and cereals, as well as specific compounds, alone or in combination. Curcumin [114], cinnamon [115,116], berberine [117,118], citrus flavonoids [119,120], quercetin [121,122], the bioactive compounds of garlic [123,124], red yeast rice [125] and neem extracts [126] have all demonstrated some activity on insulin sensitivity, but studies are usually of poor quality and very few received extensive validation, although supported by systematic reviews [119]. They may be included in dietary recommendations but should never replace pharmacologic treatment.

Resveratrol, a polyphenol present in plants such as grapes and nuts and mainly in derivatives (wine), merits a specific citation [127,128,129]. A recent Cochrane review identified three RCTs with a total of 50 participants who received graded doses of daily oral resveratrol for 4–5 weeks vs. placebo. Studies had a low risk of bias, but the analysis did not demonstrate any significant effect on glucose and HbA1c levels, with the limit of a short observation period. The authors found eight more ongoing RCTs with approximately 800 participants, likely to contribute more solid results [128]. Clinical studies in patients with insulin resistance and NAFLD have shown promising results [130], but even moderate alcohol intake is questioned in these patients due to the negative effects of alcohol on hepatic and extrahepatic cancers, which outweigh the possible beneficial effects on the cardiovascular system, largely derived from retrospective studies [131]. Finally, alcohol provides extra calories that should be considered in patients on dietary restriction, the pivotal intervention to reduce body weight and NAFLD burden.

Probiotics and/or prebiotics could be a promising approach to improve insulin sensitivity by modification of gut microbiota. Clinical data are specifically referred to gestational diabetes [132,133]; in these women four high-quality RCTs (288 participants) showed that treatment was associated with a significant reduction in insulin resistance (HOMA-IR: −0.69%; 95% CI −1.24, −0.14, *p* = 0.01), not in fasting glucose (−0.13 mmol/L; 95% CI −0.32, 0.06, *p* = 0.18) or LDL-cholesterol (−0.16 mmol/L; 95% CI −0.45, 0.13, *p* = 0.67) [133]. In the general diabetes population, the most recent review identified 38 studies totalling 2086 participants fitting pre-defined criteria to be included in a meta-analysis [134]. Overall, the use of prebiotics, probiotics or synbiotics reduced fasting glucose (−0.58 mmol/L; 95% CI −0.86, −0.30; *p* < 0.01), total cholesterol (−0.14 mmol/L; 95% CI −0.26, −0.02, *p* = 0.02) and triglyceride levels (−0.11 mmol/L; 95% CI −0.20, −0.02, *p* = 0.01) and increased HDL-cholesterol (0.04 mmol/L; 95% CI 0.01, 0.07, *p* < 0.01), but failed to reach the significance threshold in HbA1c (−2.17 mmol/mol; 95% CI, −4.37 to 0.03; *p* = 0.05) and had no effect on LDL-cholesterol [134].

Fructans are compounds acting as prebiotics, i.e., non-digestible food ingredients neither metabolized nor absorbed while passing through the upper gastrointestinal tract and fermented by bacteria in the colon. They include fructo-oligosaccharides, galacto-oligosaccharides, lactulose and large polysaccharides (inulin, resistant starches, cellulose, hemicellulose, pectin and gum) [135,136]. Diets rich in fructans might improve glucose metabolism in T2D also via decreased intake and intestinal absorption of food, adding to modifications of gut microbiota [137,138]. A systematic review with meta-analysis of 25 studies did not provide evidence for a beneficial effect on BMI, but inulin-type carbohydrate supplementation reduced fasting glucose (−16.4 mg/dL; 95% CI, −17.6 to −15.2), HbA1c (−0.58%; 95% CI, −0.78 to −0.39), and HOMA-IR (−0.99%; 95% CI, −1.76 to −0.2). However, a large heterogeneity was demonstrated, raising doubts on data validity [139].

### 4.5. Other Micronutrients

#### 4.5.1. Zinc

Zinc deficiency is common in T2D [140], likely as an effect of both hyperzincuria [141] and reduced intestinal absorption [142], resulting in insulin resistance [143]. Its antioxidant role further strengthens the importance of zinc levels for diabetes control and the prevention of microvascular complications [144].

In the clinical setting, a systematic review with meta-analysis of 12 studies in T2D patients showed that zinc supplementation resulted in a significant reduction of fasting blood glucose (pooled mean difference, −18.1 mg/dL; 95% CI −33.8 to −2.41) and HbA1c (−0.54 %; 95%CI, –0.86 to –0.21), accompanied by a systematic reduction of total and LDL-cholesterol levels [145]. Among diabetes-related complications, zinc supplementation was shown to reduce lipoperoxidation [146] and to decrease urinary albumin excretion, independently of glucose control [147,148]. However, a few studies failed to demonstrate any positive effect of zinc supplementation in the metabolic control of T2D patients [146], also in the presence of long-term supplementation and low zinc levels at baseline [149]. Zinc supplementation might prove useful only in specific settings. In zinc-deficient patients with cirrhosis, independently of diabetes status, zinc treatment (zinc sulfate, 200 mg three times per day) was associated with improved non-insulin-mediated glucose disposal (so-called glucose effectiveness) [150], as well as improved alanine stimulated urea synthesis rate, a measure of amino acid utilization in tissues [151], also resulting in decreased ammonia levels and improved mental state. All these complementary effects might be important in subjects with T2D progressed to NAFLD-cirrhosis [152].

No relevant side effects of zinc supplements have been reported in chronic diseases [153].

#### 4.5.2. Chromium

A possible role of deficient chromium levels as risk factor T2D has long been suggested based on its insulin-sensitising activity, but the effects on human disease remain uncertain. In a large case-control study involving 4443 Chinese individuals (nearly half with either newly diagnosed T2D or newly diagnosed pre-diabetes), plasma chromium levels were approximately 10% lower in the T2D and pre-diabetes groups vs. controls, and the risk of T2D and pre-diabetes decreased across quartiles of chromium [154]. This evidence fits with smaller studies reporting decreased chromium levels and/or increased chromium excretion in T2D [141,155].

The effects of chromium supplementation have been tested in multiple review articles with pooled analysis or metanalysis [156,157,158,159]. Based on 25 RCTs of chromium supplementation, Suksomboon et al., concluded for positive effects of chromium supplementation on glucose control in patients with diabetes, with no increased risks of adverse events compared with placebo [156]. On the contrary, Yin et al., in a meta-analysis of 14 trials (875 participants, mean age range: 30 to 83 years old, 8 to 24 weeks of follow-up) did not demonstrate any significant effect of chromium, either as Cr chloride, or Cr picolinate, or Cr yeast) on HbA1c levels [157]. In a review limited to patients with T2D, very few studies reached clinically meaningful goals, defined as fasting plasma glucose (FPG) ≤7.2 mmol/dL, a decline in HbA1c to values ≤7%, or a decrease of ≥0.5% in baseline levels [158]. Finally, in the most recent and largest analysis in T2D (28 studies, 1295 participants, heterogeneous chromium supplements with daily intake ranging up to 3000 µg for 6–24 weeks), the authors concluded for a positive effect of Cr supplements on glucose metabolism [159] and include chromium supplements into the treatment of T2D [159], despite uncertainty about long-term use. Treatment reduced fasting glucose (WMD, −0.99 mmol/L; 95% CI, −1.72 to −0.25), HbA1c (WMD, −0.54 %; 95% CI, −0.82 to −0.25), triglycerides and increased HDL-cholesterol. The effects were mainly reported using both chloride and picolinate formulations and were independent of treatment duration.

#### 4.5.3. Magnesium

Insulin modulates the shift of magnesium from extracellular to intracellular space; in turn, intracellular Mg^2+^ concentration modulates insulin action, as well as blood pressure [160]; thus, low magnesium induces insulin resistance, and insulin resistance further decreases magnesium levels [161]. In the past 20 years, several epidemiological and clinical studies have demonstrated the protective role of magnesium on the risk of diabetes. In U.S. women aged ≥45 years (Women’s Health Study) with no previous history of T2D, an inverse association was found between dietary magnesium and incident T2D, which was significant among women with increasing grades of overweight/obesity (P for trend, 0.02). It was associated with a progressive decline of insulin levels (P for trend, 0.03) [162]. Data were confirmed in 1122 individuals (20–65 years of age) enrolled between 1996 and 1997 and re-examined about 10 years later. The relative risk of new-onset prediabetes and T2D were increased in the presence of low magnesium levels at baseline [163].

Oral magnesium supplementation in subjects with T2D and low magnesium levels have been reported to improve insulin sensitivity and metabolic control [164,165,166]. In a meta-analysis of 40 prospective cohort studies enrolling more than 1 million participants and follow-up periods ranging from 4 to 30 years, dietary magnesium intake was associated with a 19% reduction in the relative risk of T2D (RR 0.81; 95% CI, 0.77–0.86 per 100 mg/day increment) [167]. In a different analysis of 28 studies involving 1694 subjects (834 in the treatment arm and 860 in the placebo arm), magnesium supplementation was demonstrated to produce favourable effects on blood glucose (WMD, −4.64 mg dL, 95% CI −7.60 to −1.68), as well as on HDL- and LDL-cholesterol, triglycerides and systolic blood pressure, also reducing cardiovascular risk [168].

Additionally, for magnesium supplements, no safety concerns have been raised; Verma and coll. argue that large trials should be performed to validate the use of magnesium supplements to prevent and treat T2D [168], but no consensus exists in the community [169].

## 5. Prevention and Treatment of Diabetes-Related Sarcopenia

Optimal energy intake, healthy food choices and sufficient protein intake, coupled with habitual physical activity, especially resistance training, are the cornerstones for metabolic control and the prevention of frailty in T2D. Despite the mounting evidence of the negative impact of sarcopenia on the natural history [170] and quality of life of T2D patients [171], there is a surprising dearth of intervention studies addressing T2D-related sarcopenia. Therefore, we must rely on findings from general intervention studies on sarcopenia and/or sarcopenic obesity.

Resistance training represents the most effective intervention for prevention and treatment and can be safely carried out even in fragile patients [172]. High protein (1.2–1.4 g/kg) hypocaloric diets—either exclusively food-based or including protein supplements, both as an adjunct to resistance training—have proven effective for preventing muscle mass loss during weight-reduction diets in women with obesity [173]. To reach the anabolic threshold, the protein supplement should be provided at meals rather than between meals in the elderly. The optimal protein dose (including food protein and proteins from supplements) should be 30–45 g of proteins per serving in the elderly [174]. However, high protein load cannot be recommended to T2D patients with chronic kidney disease (CKD) [175].

Whey proteins, rich in the anabolic amino acid leucine, represent the most frequently used protein supplements. Additionally, BCAA supplement or the leucine metabolite β-hydroxy-β-methyl butyrate have been proposed. These supplements are generally ineffective as sole treatment in patients without diabetes [173,176,177] and must be added to resistance training to improve already-established sarcopenia (associated or not to obesity). Leucine has strong insulinotropic properties, and leucine-rich supplements may increase the availability of amino acids for protein synthesis and reduce protein breakdown in the muscle, at the same time enhancing glucose disposal and glycaemic control, but solid data are lacking [178]. A noteworthy issue is that BCAA treatment has proven effective both in preventing and in improving sarcopenia in patients with liver cirrhosis, also independently of physical exercise/resistance training [179,180].

Finally, vitamin D was also proposed as a nutritional supplement to control sarcopenia. The activation of the vitamin D receptor present in muscle cells promotes their differentiation, proliferation and hypertrophy. Vitamin D deficiency is associated with reduced muscle mass and strength in the elderly [181], and vitamin D supplementation increased muscle strength, particularly in vitamin D-deficient cases and in the elderly [181]. Data were not confirmed by a Cochrane review in patients with liver disease; no data are available in T2D [182] and trials are eagerly warranted.

## 6. Management of Other Comorbidity in Patients with T2D

### 6.1. Cirrhosis

Nutrition therapy in cirrhosis has already been discussed in this Special Issue of Nutrients. Nonetheless, its association with T2D deserves a special focus considering the high prevalence—up to two-thirds of patients with cirrhosis listed for liver transplantation have T2D [183]—and its importance as a risk factor for the development of complications (ascites, hepatic encephalopathy, bacterial infections, renal insufficiency, hepatocellular carcinoma) [184]. Nutrition treatment becomes extremely challenging since additional determinants of malnutrition may be present, including reduced food intake and/or defective absorption of nutrients and impaired albumin synthesis. Sarcopenia—accelerated by upregulation of myostatin due to hyperammonaemia—becomes a predictor of morbidity and mortality, aggravated by obesity (sarcopenic obesity) [185,186], and is difficult to treat. Bariatric surgery is frequently contraindicated [187]; also pharmacologic treatment with GLP-1 agonists favouring weight loss [188], such as liraglutide, may be contraindicated by the presence of varices at risk of bleeding [189], and dietary treatment remains the sole possibility.

Unfortunately, there are no specific guidelines for the nutritional treatment of T2D associated with cirrhosis, and individualized, structured nutritional programs are suggested to accomplish the need for restriction of sodium and fluids [190]. Due to the accelerated depletion of glycogen stores, it is important to provide frequent (3 to 5) meals containing carbohydrates, plus a late evening carbohydrate snack to prevent muscle protein catabolism [191,192].

Protein restriction is not systematically advocated, as these patients usually tolerate a normal protein intake. Besides hypoalbuminemia, potentially requiring a higher protein intake, albumin glycation is present in T2D [193]. The structurally damaged albumin molecule is also dysfunctional, and albumin administration may be required to reduce ascites. Although the specific indications for use are clearly defined by international guidelines [194], albumin is frequently administered outside evidence-based indications, including nutritional support [195]. At present, no studies showed a direct link between albumin administration and nutritional correction in decompensated cirrhosis; it can only be hypothesized that the clinical improvement seen with long-term albumin treatment could indirectly improve the nutritional status through different mechanisms, which include the control/resolution of ascites and whole body edema, or the reduction of systemic inflammation [196].

### 6.2. Renal Failure

In T2D patients with CKD, protein restriction may be advised; low protein diets (daily protein intake reduced to 0.8 g/kg b.w.) showed a beneficial impact on the trajectory of renal function leading to an attenuation in the progression of CKD and delayed initiation of dialysis treatment, an important goal for patients [197,198,199]. However, protein restriction may worsen sarcopenia and should be limited as long as possible. According to the National Kidney Foundation/Kidney Disease Outcomes Quality Initiative (NKF-KDOQI) Guidelines, protein intake must actually be increased up to 1.2 g/kg body in patients undergoing maintenance dialysis due to important additional amino acid losses occurring in dialysate [200,201].

Different sources of dietary protein may have a different impact on CKD-related complications; meat intake increases the production of nitrogenous end products, worsens uraemia and may increase the risk of constipation with consequent hyperkalaemia associated with the low fibre intake [199]. A predominantly plant-based diet, fibre-rich and low in protein content (0.6–0.8 g/kg/day), can produce favourable changes in the intestinal microbiome, thus modulating the generation of uremic toxins and slowing down the progression of CKD, finally reducing cardiovascular risk [202]. Carbohydrates from sugars should be limited to less than 10% of the energy intake [203], and saturated fatty acids, trans fats, and cholesterol should be replaced by polyunsaturated and monounsaturated fats, associated with more favourable outcomes [204]. Dietary sodium restriction should be considered, but a deficient sodium intake (to less than 1.5–2.0 g/day) carries the risk of hyponatremia, leading to reduced insulin sensitivity and prediabetes [205]. T2D patients with advanced CKD progressing to end-stage renal disease may be prone to the “burnt-out diabetes” phenomenon (i.e., spontaneous resolution of hyperglycaemia and frequent hypoglycaemic episodes); further studies in this frail population in chronic hemodialysis treatment are particularly needed to determine the safety and the effectiveness of dietary manipulations [206].

## 7. Conclusions

T2D is the paradigm of conditions where genetic, behavioural and individual factors drive disease occurrence and severity. Despite decades of epidemiological studies and randomized trials, several unmet needs remain (Table 4). The goal of optimal nutritional approach is to maintain or regain a body weight within the normal range, providing adequate intake of macronutrients and micronutrients to reduce the risk of sarcopenia. Various dietary approaches have been proposed to improve outcome, with the Mediterranean diet supported by solid evidence. However, as long-term adherence is the main goal to be achieved, the dietary plan and the calorie restriction that patients feel confident to maintain life-long should always be preferred. At present, supplementation with inositols, vitamin D and micronutrients (zinc, chromium, magnesium) is not systematically suggested, but might be considered in individual patients.

Although advances in nutrigenomics and metabolomics offer the rationale for tailored precision medicine, a personalized meal plan, supported by continuous dietary counselling by registered dietitians remains at present the key strategy for long-term success in weight and glycaemic control [37], particularly in individual high-risk cases [38].

## Figures and Tables

**Table 1:** Comparison of strategies and goals of different dietary interventions.

	Dietary Prescription	Dietary Counseling	Behaviour Therapy
Dietary program	• Based on rigid meal prescription (food weight, substitution schedule)	• Proposed food choices with templates of daily meals	• Food choices proposed within the frame of a healthy diet
Patient role	• Passive adherence to the prescriptive plans	• Acquires competence in healthy diet strategies	• Meals and physical activity planned according to personal preferences
Role of therapist	• Active–gives the solution • The planned calorie intake is mandatory for patients	• Provides education on healthy lifestyles • Helps in identifying best practices according to patients’ preferences and individual status	• Communicates empathically • Supports patients’ activities, success and failures to stimulate self-efficacy • Helps in identifying obstacles and presents possible solutions
Treatment goals	• Strong focus on weight loss or HbA1c targets	• Set realistic expectations and acceptable body weight	• Behavioural changes are the main targets, independent of the amount of weight loss
Temporal terms	• Usually limited to weeks, with frequent changes	• Life-long adherence to healthy lifestyles	• Life-long adherence to healthy lifestyles
Additional components	-------------	• Integration of dietary and physical activity counselling	• Generation of a mindset favouring lifestyle targets
Psychological support	-------------	• Support by family, significant others, both on food choices and in habitual physical activity	• Implementation of a pro-active problem solving • Stimulus control aimed at modifying the environment • Strategies of cognitive restructuring to address dysfunctional thoughts

Note that enrolment into counselling and behaviour therapy may be facilitated by motivational interviewing. Treatment may be provided either in individual or in group settings; group strategies are likely to enhance the coping skills of the participants, via relational and interpersonal communication with people experiencing similar difficulties.

**Table 2:** Summary of nutritional recommendations for type 2 diabetes, as derived from international guidelines.

Nutrients	Recommendations
Calorie intake	• Reduce energy intake in all individuals with overweight/obesity (calorie deficit of 250–500 kcal/day) to promote weight loss (0.5–1.0 kg/week) to a final body weight within the normal range
Macronutrient distribution	• There is insufficient evidence to recommend specific macronutrient distribution, but a moderate carbohydrate reduction might favour glucose control and promote a moderate weight loss
Carbohydrates	• Prefer low glycaemic index foods (whole grains, fruits, legumes, green salad with olive oil dressing and most vegetables). Limit refined carbohydrates (pasta, white bread, rice, white potatoes, etc.)
Sugars	• Limit intake of sucrose-containing foods and sugary drinks • Prefer non-nutritive sweeteners as substitutes of sugar • Low calorie or unsweetened beverages should be preferred, but their consumption limited
Fibers	• 30–50 g/day of dietary fibres (at least one-third of soluble origin)
Proteins	• As in the general population, 1.0–1.5 g/kg ideal body weight • Reduce protein intake to 0.8 g kg/b.w. or lower in patients with chronic kidney disease
Fats	• As in the general population, 20–35% of total kcal/day • Avoid trans-fatty acids and limit saturated fatty acids (SFAs) to 7–9%. Increase foods enriched in long-chain omega-3 polyunsaturated fatty acids (PUFAs) and monounsaturated fatty acids (MUFAs).
Micronutrients & Vitamins	• Correct micronutrient and vitamin deficiencies • Consider vitamin supplementation (B-group vitamins or folic acid) in metformin-treated patients
Sodium	• Limited as in the general population; consider additional limitations in those with hypertension
Alcohol	• Limited as in the general population
Dietary pattern	• Favour a dietary model based on Mediterranean-style

**Table 3:** Putative mechanism(s) responsible for the beneficial effects of nutrition supplements and micronutrients on diabetes risk and glycaemic control.

Product	Mechanism of Action
Inositols [69,70,71]	Myo-inositol (MI) and D-chiro-inositol (DCI) act as insulin second messengers. MI takes part in cellular glucose uptake and is high in the brain and heart, where high rates of glucose utilization occur MI prevents the release of free fatty acids from adipose tissues; on the contrary, DCI is involved in glycogen storage, being elevated in the liver, muscle, and fat tissue DCI may be preferred to MI to restore insulin sensitivity and glycogen synthesis because it bypasses the defective epimerization of MI to DCI in the presence of insulin resistance
Vitamin D [72,73,74,75]	Serum levels of 25(OH)D are significantly lower in patients with T2D compared with values measured in healthy people, with a negative correlation with HOMA-IR and adipokines Direct effect on insulin secretion, mediated by nuclear vitamin D receptors also present in pancreatic β-cells, but the effects on insulin sensitivity have long been conflicting Vitamin D deficiency is associated with vascular inflammatory responses by promoting the secretion of inflammatory cytokines
Niacin [76]	This compound mediates hundreds of oxidation-reduction redox reactions, which are essential sources of energy for a myriad of cellular functions, finally known to improve lipid profile and to reduce cardiovascular risk Restoration of C-peptide release, but unexplained negative results on glycaemic control
Resveratrol [77,78]	Activator of the sirtuin pathway, regulating several cellular functions related to metabolism, oxidation, and aging Anti-oxidant activity
Pre/probiotics [79,80,81,82,83]	Effects on insulin sensitivity by modification of gut microbiota
Zinc [84,85]	Participation in insulin synthesis, storage, crystallization, and secretion in the pancreatic β-cell, as in well as in insulin action and translocation inside the cells Stimulation of insulin sensitivity through the activation of the phosphoinositol-3-kinase/protein kinase B cascade. Stimulation of glucose uptake in insulin-independent tissues (insulin-mimetic action) Suppression of proinflammatory cytokines (interleukin-1β and nuclear factor kβ), thus avoiding β-cells death and protecting insulin
Chromium [86,87]	Effects on insulin signalling Insulin sensitising activity in experimental animals
Magnesium [88,89]	Possible effects of Mg^2+^ deficiency on the tricarboxylic acid cycle, increasing the risk of hyperinsulinemia and insulin resistance Modulation of insulin action and oxidative glucose metabolism Alteration of lipid metabolism and the antioxidant system

Abbreviations: HOMA-IR, homeostasis model assessment of insulin resistance; IL, interleukin; TNF, tumor-necrosis factor.

**Table 4:** Principal unmet needs for optimal nutritional treatment of patients with type 2 diabetes.

Target	Unmet Needs
Weight control	Define the best dietary plan to support weight loss and weight loss maintenance Define the role of psychological support in individual, difficult cases Define the role of e-health technology and individual apps to improve long-term adherence to dietary recommendations
Prevention and treatment of sarcopenia	Define the optimal protein intake to prevent sarcopenia How to integrate physical activity in the daily life of frail patients Assess the relative role of resistance vs. aerobic exercise
Vitamins and nutritional supplements	Are they really needed (if, when, to whom)? Who should be screened for micronutrient deficit Define the role of nutritional supplements in the prevention of sarcopenia Define the possible interaction of vitamins and nutrients with drug treatment

## Data Availability

Not applicable.

## References

[1] International Diabetes Federation (2019). IDF Diabetes Atlas.

[2] American Diabetes Association (2021). 3. Prevention or delay of type 2 diabetes: Standards of medical care in Diabetes-2021. Diabetes Care.

[3] Cruz-Jentoft A.J., Landi F., Schneider S.M., Zuniga C., Arai H., Boirie Y., Chen L.K., Fielding R.A., Martin F.C., Michel J.P. (2014). Prevalence of and interventions for sarcopenia in ageing adults: A systematic review. Report of the International Sarcopenia Initiative (EWGSOP and IWGS). Age Ageing.

[4] Kalyani R.R., Corriere M., Ferrucci L. (2014). Age-related and disease-related muscle loss: The effect of diabetes, obesity, and other diseases. Lancet Diabetes Endocrinol..

[5] American Diabetes Association (2021). 5. Facilitating behavior change and well-being to improve health outcomes: Standards of medical care in Diabetes-2021. Diabetes Care.

[6] American Diabetes Association (2021). 8. Obesity management for the treatment of type 2 diabetes: Standards of medical care in Diabetes-2021. Diabetes Care.

[7] Sievenpiper J.L., Chan C.B., Dworatzek P.D., Freeze C., Williams S.L. (2018). Diabetes Canada Clinical Practice Guidelines Expert Committee. Nutrition therapy. Can. J. Diabetes.

[8] Franz M.J., MacLeod J., Evert A., Brown C., Gradwell E., Handu D., Reppert A., Robinson M. (2017). Academy of Nutrition and Dietetics Nutrition Practice Guideline for type 1 and type 2 diabetes in adults: Systematic review of evidence for medical nutrition therapy effectiveness and recommendations for integration into the nutrition care process. J. Acad. Nutr. Diet..

[9] Associazione Medici Diabetologi (AMD), Società Italiana di Diabetologia (SID) (2018). Standard Italiani per la Cura del Diabete.

[10] Leslie W.S., Ford I., Sattar N., Hollingsworth K.G., Adamson A., Sniehotta F.F., McCombie L., Brosnahan N., Ross H., Mathers J.C. (2016). The Diabetes Remission Clinical Trial (DiRECT): Protocol for a cluster randomised trial. BMC Fam. Pract..

[11] Dalle Grave R., Calugi S., Centis E., Marzocchi R., El Ghoch M., Marchesini G. (2010). Lifestyle modification in the management of the metabolic syndrome: Achievements and challenges. Diabetes Metab. Syndr. Obes. Targets Ther..

[12] National Institutes of Health (1980). Successful diet and exercise therapy is conducted in Vermont for “diabesity”. JAMA.

[13] Astrup A., Finer N. (2000). Redefining type 2 diabetes: ‘Diabesity’ or ‘obesity dependent diabetes mellitus’?. Obes. Rev..

[14] Zimmet P. The Negev desert, migration, islands and diabesity: Genes and environment clashing. Proceedings of the Cohen Memorial Lecture 36th Annual Meeting of the European Association for the Study of Diabetes.

[15] Flegal K.M., Shepherd J.A., Looker A.C., Graubard B.I., Borrud L.G., Ogden C.L., Harris T.B., Everhart J.E., Schenker N. (2009). Comparisons of percentage body fat, body mass index, waist circumference, and waist-stature ratio in adults. Am. J. Clin. Nutr..

[16] Wilson M.M., Morley J.E. (2003). Invited review: Aging and energy balance. J. Appl. Physiol..

[17] The Lancet Diabetes Endocrinology (2014). Sarcopenia: A fate worth challenging. Lancet Diabetes Endocrinol..

[18] Marini E., Buffa R., Saragat B., Coin A., Toffanello E.D., Berton L., Manzato E., Sergi G. (2012). The potential of classic and specific bioelectrical impedance vector analysis for the assessment of sarcopenia and sarcopenic obesity. Clin. Interv. Aging.

[19] Buffa R., Saragat B., Cabras S., Rinaldi A.C., Marini E. (2013). Accuracy of specific BIVA for the assessment of body composition in the United States population. PLoS ONE.

[20] Cruz-Jentoft A.J., Bahat G., Bauer J., Boirie Y., Bruyere O., Cederholm T., Cooper C., Landi F., Rolland Y., Sayer A.A. (2019). Sarcopenia: Revised European consensus on definition and diagnosis. Age Ageing.

[21] Izzo A., Massimino E., Riccardi G., Della Pepa G. (2021). A narrative review on sarcopenia in type 2 diabetes mellitus: Prevalence and associated factors. Nutrients.

[22] Anagnostis P., Gkekas N.K., Achilla C., Pananastasiou G., Taouxidou P., Mitsiou M., Kenanidis E., Potoupnis M., Tsiridis E., Goulis D.G. (2020). Type 2 diabetes mellitus is associated with increased risk of sarcopenia: A systematic review and meta-analysis. Calcif. Tissue Int..

[23] Solanki J.D., Makwana A.H., Mehta H.B., Gokhale P.A., Shah C.J. (2015). Body composition in type 2 diabetes: Change in quality and not just quantity that matters. Int. J. Prev. Med..

[24] Bawadi H., Alkhatib D., Abu-Hijleh H., Alalwani J., Majed L., Shi Z. (2020). Muscle strength and glycaemic control among patients with type 2 diabetes. Nutrients.

[25] Lombardo M., Padua E., Campoli F., Panzarino M., Mindrescu V., Annino G., Iellamo F., Bellia A. (2021). Relative handgrip strength is inversely associated with the presence of type 2 diabetes in overweight elderly women with varying nutritional status. Acta Diabetol..

[26] Leenders M., Verdijk L.B., van der Hoeven L., Adam J.J., van Kranenburg J., Nilwik R., van Loon L.J. (2013). Patients with type 2 diabetes show a greater decline in muscle mass, muscle strength, and functional capacity with aging. J. Am. Med. Dir. Assoc..

[27] Veronese N., Stubbs B., Punzi L., Soysal P., Incalzi R.A., Saller A., Maggi S. (2019). Effect of nutritional supplementations on physical performance and muscle strength parameters in older people: A systematic review and meta-analysis. Ageing Res. Rev..

[28] Petersen K.F., Dufour S., Savage D.B., Bilz S., Solomon G., Yonemitsu S., Cline G.W., Befroy D., Zemany L., Kahn B.B. (2007). The role of skeletal muscle insulin resistance in the pathogenesis of the metabolic syndrome. Proc. Natl. Acad. Sci. USA.

[29] Haedersdal S., Lund A., Knop F.K., Vilsboll T. (2018). The role of glucagon in the pathophysiology and treatment of type 2 diabetes. Mayo Clin. Proc..

[30] Taylor R. (2013). Type 2 diabetes: Etiology and reversibility. Diabetes Care.

[31] Galsgaard K.D., Winther-Sorensen M., Orskov C., Kissow H., Poulsen S.S., Vilstrup H., Prehn C., Adamski J., Jepsen S.L., Hartmann B. (2018). Disruption of glucagon receptor signaling causes hyperaminoacidemia exposing a possible liver-alpha-cell axis. Am. J. Physiol. Endocrinol. Metab..

[32] Wewer Albrechtsen N.J., Faerch K., Jensen T.M., Witte D.R., Pedersen J., Mahendran Y., Jonsson A.E., Galsgaard K.D., Winther-Sorensen M., Torekov S.S. (2018). Evidence of a liver-alpha cell axis in humans: Hepatic insulin resistance attenuates relationship between fasting plasma glucagon and glucagonotropic amino acids. Diabetologia.

[33] Younossi Z.M., Golabi P., de Avila L., Paik J.M., Srishord M., Fukui N., Qiu Y., Burns L., Afendy A., Nader F. (2019). The global epidemiology of NAFLD and NASH in patients with type 2 diabetes: A systematic review and meta-analysis. J. Hepatol..

[34] Felig P. (1975). Amino acid metabolism in man. Annu. Rev. Biochem..

[35] Forlani G., Vannini P., Marchesini G., Zoli M., Ciavarella A., Pisi E. (1984). Insulin-dependent metabolism of branched-chain amino acids in obesity. Metabolism.

[36] Evert A.B., Dennison M., Gardner C.D., Garvey W.T., Lau K.H.K., MacLeod J., Mitri J., Pereira R.F., Rawlings K., Robinson S. (2019). Nutrition therapy for adults with diabetes or prediabetes: A consensus report. Diabetes Care.

[37] Forouhi N.G., Misra A., Mohan V., Taylor R., Yancy W. (2018). Dietary and nutritional approaches for prevention and management of type 2 diabetes. BMJ.

[38] Wang D.D., Hu F.B. (2018). Precision nutrition for prevention and management of type 2 diabetes. Lancet Diabetes Endocrinol..

[39] Ojo O. (2019). Dietary intake and type 2 diabetes. Nutrients.

[40] Meng Y., Bai H., Wang S., Li Z., Wang Q., Chen L. (2017). Efficacy of low carbohydrate diet for type 2 diabetes mellitus management: A systematic review and meta-analysis of randomized controlled trials. Diabetes Res. Clin. Pract..

[41] Ojo O., Ojo O.O., Adebowale F., Wang X.H. (2018). The effect of dietary glycaemic index on glycaemia in patients with type 2 diabetes: A systematic review and meta-analysis of randomized controlled trials. Nutrients.

[42] Hamdy O., Barakatun-Nisak M.Y. (2016). Nutrition in Diabetes. Endocrinol. Metab. Clin. N. Am..

[43] Vogtschmidt Y.D., Raben A., Faber I., de Wilde C., Lovegrove J.A., Givens D.I., Pfeiffer A.F.H., Soedamah-Muthu S.S. (2021). Is protein the forgotten ingredient: Effects of higher compared to lower protein diets on cardiometabolic risk factors. A systematic review and meta-analysis of randomised controlled trials. Atherosclerosis.

[44] Xue L., Yin R., Howell K., Zhang P. (2021). Activity and bioavailability of food protein-derived angiotensin-I-converting enzyme-inhibitory peptides. Compr. Rev. Food Sci. Food Saf..

[45] Qian F., Korat A.A., Malik V., Hu F.B. (2016). Metabolic effects of monounsaturated fatty acid-enriched diets compared with carbohydrate or polyunsaturated fatty acid-enriched diets in patients with type 2 diabetes: A systematic review and meta-analysis of randomized controlled trials. Diabetes Care.

[46] Georgoulis M., Kontogianni M.D., Yiannakouris N. (2014). Mediterranean diet and diabetes: Prevention and treatment. Nutrients.

[47] Mesías M., Seiquer I., Delgado-Andrade C., Preedy V., Watson R. (2020). The Mediterranean diet and mineral composition. The Mediterranean Diet: An Evidence-Based Approach.

[48] U.S. Department of Health and Human Services, U.S. Department of Agriculture (2015). 2015–2020 Dietary Guidelines for Americans, 8th ed. http://health.gov/dietaryguidelines/2015/guidelines.

[49] Campbell A.P. (2017). DASH eating plan: An eating pattern for diabetes management. Diabetes Spectr..

[50] Chester B., Babu J.R., Greene M.W., Geetha T. (2019). The effects of popular diets on type 2 diabetes management. Diabetes Metab. Res. Rev..

[51] Tuomilehto J., Lindstrom J., Eriksson J.G., Valle T.T., Hamalainen H., Ilanne-Parikka P., Keinanen-Kiukaanniemi S., Laakso M., Louheranta A., Rastas M. (2001). Prevention of type 2 diabetes mellitus by changes in lifestyle among subjects with impaired glucose tolerance. N. Engl. J. Med..

[52] The Diabetes Prevention Program Research Group (2002). The Diabetes Prevention Program (DPP): Description of lifestyle intervention. Diabetes Care.

[53] Wadden T.A., West D.S., Delahanty L., Jakicic J., Rejeski J., Williamson D., Berkowitz R.I., Kelley D.E., Tomchee C., The Look Ahead Research Group (2006). The Look AHEAD study: A description of the lifestyle intervention and the evidence supporting it. Obesity.

[54] Wing R.R., Bolin P., Brancati F.L., Bray G.A., Clark J.M., Coday M., Crow R.S., Curtis J.M., Egan C.M., Espeland M.A. (2013). Cardiovascular effects of intensive lifestyle intervention in type 2 diabetes. N. Engl. J. Med..

[55] Petroni M.L., Caletti M.T., Calugi S., Dalle Grave R., Marchesini G. (2017). Long-term treatment of severe obesity: Are lifestyle interventions still an option?. Expert Rev. Endocrinol. Metab..

[56] Ikramuddin S., Korner J., Lee W.J., Thomas A.J., Connett J.E., Bantle J.P., Leslie D.B., Wang Q., Inabnet W.B., Jeffery R.W. (2018). Lifestyle Intervention and medical management with vs without Roux-en-Y gastric bypass and control of hemoglobin A1c, LDL cholesterol, and systolic blood pressure at 5 years in the Diabetes Surgery Study. JAMA.

[57] Lean M.E., Leslie W.S., Barnes A.C., Brosnahan N., Thom G., McCombie L., Peters C., Zhyzhneuskaya S., Al-Mrabeh A., Hollingsworth K.G. (2018). Primary care-led weight management for remission of type 2 diabetes (DiRECT): An open-label, cluster-randomised trial. Lancet.

[58] Lean M.E.J., Leslie W.S., Barnes A.C., Brosnahan N., Thom G., McCombie L., Peters C., Zhyzhneuskaya S., Al-Mrabeh A., Hollingsworth K.G. (2019). Durability of a primary care-led weight-management intervention for remission of type 2 diabetes: 2-year results of the DiRECT open-label, cluster-randomised trial. Lancet Diabetes Endocrinol..

[59] Taylor R., Al-Mrabeh A., Zhyzhneuskaya S., Peters C., Barnes A.C., Aribisala B.S., Hollingsworth K.G., Mathers J.C., Sattar N., Lean M.E.J. (2018). Remission of human type 2 diabetes requires decrease in liver and pancreas fat content but Is dependent upon capacity for beta cell recovery. Cell Metab..

[60] Garcia-Molina L., Lewis-Mikhael A.M., Riquelme-Gallego B., Cano-Ibanez N., Oliveras-Lopez M.J., Bueno-Cavanillas A. (2020). Improving type 2 diabetes mellitus glycaemic control through lifestyle modification implementing diet intervention: A systematic review and meta-analysis. Eur. J. Nutr..

[61] Grajower M.M., Horne B.D. (2019). Clinical management of intermittent fasting in patients with diabetes mellitus. Nutrients.

[62] Rajpal A., Ismail-Beigi F. (2020). Intermittent fasting and ‘metabolic switch’: Effects on metabolic syndrome, prediabetes and type 2 diabetes. Diabetes Obes. Metab..

[63] Horne B.D., Grajower M.M., Anderson J.L. (2020). Limited evidence for the health effects and safety of intermittent fasting among patients with type 2 diabetes. JAMA.

[64] Higgins J.P. (2016). Smartphone applications for patients’ health and fitness. Am. J. Med..

[65] Toro-Ramos T., Michaelides A., Anton M., Karim Z., Kang-Oh L., Argyrou C., Loukaidou E., Charitou M.M., Sze W., Miller J.D. (2020). Mobile delivery of the Diabetes Prevention Program in people with prediabetes: Randomized controlled trial. JMIR mHealth uHealth.

[66] Stein N., Brooks K. (2017). A fully automated conversational artificial intelligence for weight loss: Longitudinal cbservational study among overweight and obese ddults. JMIR Diabetes.

[67] Chin S.O., Keum C., Woo J., Park J., Choi H.J., Woo J.T., Rhee S.Y. (2016). Successful weight reduction and maintenance by using a smartphone application in those with overweight and obesity. Sci. Rep..

[68] Mazzotti A., Caletti M.T., Brodosi L., Di Domizio S., Forchielli M.L., Petta S., Bugianesi E., Bianchi G., Marchesini G. (2018). An internet-based approach for lifestyle changes in patients with NAFLD: Two-year effects on weight loss and surrogate markers. J. Hepatol..

[69] Nestler J.E., Unfer V. (2015). Reflections on inositol(s) for PCOS therapy: Steps toward success. Gynecol. Endocrinol..

[70] Unfer V., Nestler J.E., Kamenov Z.A., Prapas N., Facchinetti F. (2016). Effects of inositol(s) in women with PCOS: A systematic review of randomized controlled trials. Int. J. Endocrinol..

[71] Larner J. (2002). D-chiro-inositol--its functional role in insulin action and its deficit in insulin resistance. Int. J. Exp. Diabetes Res..

[72] Walters M.R. (1992). Newly identified actions of the vitamin D endocrine system. Endocr. Rev..

[73] Pittas A.G., Harris S.S., Stark P.C., Dawson-Hughes B. (2007). The effects of calcium and vitamin D supplementation on blood glucose and markers of inflammation in nondiabetic adults. Diabetes Care.

[74] Wang W., Zhang J., Wang H., Wang X., Liu S. (2019). Vitamin D deficiency enhances insulin resistance by promoting inflammation in type 2 diabetes. Int J. Clin. Exp. Pathol.

[75] Chiu K.C., Chu A., Go V.L., Saad M.F. (2004). Hypovitaminosis D is associated with insulin resistance and beta cell dysfunction. Am. J. Clin. Nutr..

[76] Montserrat-de la Paz S., Bermudez B., Naranjo M.C., Lopez S., Abia R., Muriana F.J. (2016). Pharmacological effects of niacin on acute hyperlipemia. Curr. Med. Chem..

[77] Cicero A.F., Tartagni E., Ertek S. (2014). Nutraceuticals for metabolic syndrome management: From laboratory to benchside. Curr. Vasc. Pharmacol..

[78] Xia N., Daiber A., Forstermann U., Li H. (2017). Antioxidant effects of resveratrol in the cardiovascular system. Br. J. Pharmacol..

[79] Al Bander Z., Nitert M.D., Mousa A., Naderpoor N. (2020). The gut microbiota and inflammation: An overview. Int. J. Environ. Res. Public Health.

[80] Salazar J., Angarita L., Morillo V., Navarro C., Martinez M.S., Chacin M., Torres W., Rajotia A., Rojas M., Cano C. (2020). Microbiota and diabetes mellitus: Role of lipid mediators. Nutrients.

[81] Tanase D.M., Gosav E.M., Neculae E., Costea C.F., Ciocoiu M., Hurjui L.L., Tarniceriu C.C., Maranduca M.A., Lacatusu C.M., Floria M. (2020). Role of gut microbiota on onset and progression of microvascular complications of type 2 diabetes (T2DM). Nutrients.

[82] Tiderencel K.A., Hutcheon D.A., Ziegler J. (2020). Probiotics for the treatment of type 2 diabetes: A review of randomized controlled trials. Diabetes Metab. Res. Rev..

[83] Woldeamlak B., Yirdaw K., Biadgo B. (2019). Role of gut microbiota in type 2 diabetes mellitus and its complications: Novel insights and potential intervention strategies. Korean J. Gastroenterol..

[84] Shan Z., Bao W., Zhang Y., Rong Y., Wang X., Jin Y., Song Y., Yao P., Sun C., Hu F.B. (2014). Interactions between zinc transporter-8 gene (SLC30A8) and plasma zinc concentrations for impaired glucose regulation and type 2 diabetes. Diabetes.

[85] Fukunaka A., Fujitani Y. (2018). Role of zinc homeostasis in the pathogenesis of diabetes and obesity. Int. J. Mol. Sci..

[86] Maret W. (2019). Chromium supplementation in human health, metabolic syndrome, and diabetes. Met. Ions Life Sci..

[87] Yang X., Li S.Y., Dong F., Ren J., Sreejayan N. (2006). Insulin-sensitizing and cholesterol-lowering effects of chromium (D-Phenylalanine)3. J. Inorg. Biochem..

[88] Feng J., Wang H., Jing Z., Wang Y., Cheng Y., Wang W., Sun W. (2020). Role of magnesium in type 2 diabetes mellitus. Biol. Trace Elem. Res..

[89] Paolisso G., Barbagallo M. (1997). Hypertension, diabetes mellitus, and insulin resistance: The role of intracellular magnesium. Am. J. Hypertens..

[90] D’Anna R., Santamaria A., Alibrandi A., Corrado F., DI Benedetto A., Facchinetti F. (2019). Myo-inositol for the prevention of gestational diabetes mellitus. A brief review. J. Nutr. Sci. Vitaminol..

[91] Formoso G., Baldassarre M.P.A., Ginestra F., Carlucci M.A., Bucci I., Consoli A. (2019). Inositol and antioxidant supplementation: Safety and efficacy in pregnancy. Diabetes Metab. Res. Rev..

[92] Guardo F.D., Curro J.M., Valenti G., Rossetti P., Di Gregorio L.M., Conway F., Chiofalo B., Garzon S., Bruni S., Rizzo G. (2019). Non-pharmacological management of gestational diabetes: The role of myo-inositol. J. Complement. Integr. Med..

[93] Tahir F., Majid Z. (2019). Inositol supplementation in the prevention of gestational diabetes mellitus. Cureus.

[94] Brown J., Crawford T.J., Alsweiler J., Crowther C.A. (2016). Dietary supplementation with myo-inositol in women during pregnancy for treating gestational diabetes. Cochrane Database Syst. Rev..

[95] Associazione Medici Diabetologi, Società Italiana di Diabetologia (2018). Position Statement: Integratori Vitaminici, Inositolo e Probiotici nelle donne con Iperglicemia in Gravidanza. https://www.siditalia.it/news/2103-17-10-2018-position-statement-amd-sid-integratori-vitaminici-inositolo-e-probiotici-nelle-donne-con-iperglicemia-in-gravidanza.edn.

[96] Omoruyi F.O., Stennett D., Foster S., Dilworth L. (2020). New frontiers for the use of IP6 and inositol combination in treating diabetes mellitus: A review. Molecules.

[97] Davinelli S., Nicolosi D., Di Cesare C., Scapagnini G., Di Marco R. (2020). Targeting metabolic consequences of insulin resistance in polycystic ovary syndrome by D-chiro-inositol and emerging nutraceuticals: A focused review. J. Clin. Med..

[98] Cheng S., Massaro J.M., Fox C.S., Larson M.G., Keyes M.J., McCabe E.L., Robins S.J., O’Donnell C.J., Hoffmann U., Jacques P.F. (2010). Adiposity, cardiometabolic risk, and vitamin D status: The Framingham Heart Study. Diabetes.

[99] Mitri J., Muraru M.D., Pittas A.G. (2011). Vitamin D and type 2 diabetes: A systematic review. Eur. J. Clin. Nutr..

[100] Ford E.S., Ajani U.A., McGuire L.C., Liu S. (2005). Concentrations of serum vitamin D and the metabolic syndrome among U.S. adults. Diabetes Care.

[101] Scragg R., Sowers M., Bell C. (2004). Serum 25-hydroxyvitamin D, diabetes, and ethnicity in the Third National Health and Nutrition Examination Survey. Diabetes Care.

[102] Eliades M., Spyrou E., Agrawal N., Lazo M., Brancati F.L., Potter J.J., Koteish A.A., Clark J.M., Guallar E., Hernaez R. (2013). Meta-analysis: Vitamin D and non-alcoholic fatty liver disease. Aliment. Pharmacol. Ther..

[103] Lee J.H., O’Keefe J.H., Bell D., Hensrud D.D., Holick M.F. (2008). Vitamin D deficiency an important, common, and easily treatable cardiovascular risk factor?. J. Am. Coll. Cardiol..

[104] Tai K., Need A.G., Horowitz M., Chapman I.M. (2008). Vitamin D, glucose, insulin, and insulin sensitivity. Nutrition.

[105] Avenell A., Cook J.A., MacLennan G.S., McPherson G.C. (2009). Vitamin D supplementation and type 2 diabetes: A substudy of a randomised placebo-controlled trial in older people (RECORD trial, ISRCTN 51647438). Age Ageing.

[106] Li X., Liu Y., Zheng Y., Wang P., Zhang Y. (2018). The effect of vitamin D supplementation on glycemic control in type 2 diabetes patients: A systematic review and meta-analysis. Nutrients.

[107] Niroomand M., Fotouhi A., Irannejad N., Hosseinpanah F. (2019). Does high-dose vitamin D supplementation impact insulin resistance and risk of development of diabetes in patients with pre-diabetes? A double-blind randomized clinical trial. Diabetes Res. Clin. Pract..

[108] Polo V., Saibene A., Pontiroli A.E. (1998). Nicotinamide improves insulin secretion and metabolic control in lean type 2 diabetic patients with secondary failure to sulphonylureas. Acta Diabetol..

[109] Xiang D., Zhang Q., Wang Y.T. (2020). Effectiveness of niacin supplementation for patients with type 2 diabetes: A meta-analysis of randomized controlled trials. Medicine.

[110] Goldie C., Taylor A.J., Nguyen P., McCoy C., Zhao X.Q., Preiss D. (2016). Niacin therapy and the risk of new-onset diabetes: A meta-analysis of randomised controlled trials. Heart.

[111] Landray M.J., Haynes R., Hopewell J.C., Parish S., Aung T., Tomson J., Wallendszus K., Craig M., Jiang L., Collins R. (2014). Effects of extended-release niacin with laropiprant in high-risk patients. N. Engl. J. Med..

[112] Swerdlow D.I., Preiss D., Kuchenbaecker K.B., Holmes M.V., Engmann J.E., Shah T., Sofat R., Stender S., Johnson P.C., Scott R.A. (2015). HMG-coenzyme A reductase inhibition, type 2 diabetes, and bodyweight: Evidence from genetic analysis and randomised trials. Lancet.

[113] Rochlani Y., Pothineni N.V., Kovelamudi S., Mehta J.L. (2017). Metabolic syndrome: Pathophysiology, management, and modulation by natural compounds. Ther. Adv. Cardiovasc. Dis..

[114] Jin T., Song Z., Weng J., Fantus I.G. (2018). Curcumin and other dietary polyphenols: Potential mechanisms of metabolic actions and therapy for diabetes and obesity. Am. J. Physiol. Endocrinol. Metab..

[115] Deyno S., Eneyew K., Seyfe S., Tuyiringire N., Peter E.L., Muluye R.A., Tolo C.U., Ogwang P.E. (2019). Efficacy and safety of cinnamon in type 2 diabetes mellitus and pre-diabetes patients: A meta-analysis and meta-regression. Diabetes Res. Clin. Pract..

[116] Yilmaz Z., Piracha F., Anderson L., Mazzola N. (2017). Supplements for diabetes mellitus: A review of the literature. J. Pharm. Pract..

[117] Perez-Rubio K.G., Gonzalez-Ortiz M., Martinez-Abundis E., Robles-Cervantes J.A., Espinel-Bermudez M.C. (2013). Effect of berberine administration on metabolic syndrome, insulin sensitivity, and insulin secretion. Metab. Syndr. Relat. Disord..

[118] Yang J., Yin J., Gao H., Xu L., Wang Y., Xu L., Li M. (2012). Berberine improves insulin sensitivity by inhibiting fat store and adjusting adipokines profile in human preadipocytes and metabolic syndrome patients. Evid. Based Complement. Alternat. Med..

[119] Gandhi G.R., Vasconcelos A.B.S., Wu D.T., Li H.B., Antony P.J., Li H., Geng F., Gurgel R.Q., Narain N., Gan R.Y. (2020). Citrus flavonoids as promising phytochemicals targeting diabetes and related complications: A systematic review of in vitro and in vivo studies. Nutrients.

[120] Al-Aubaidy H.A., Dayan A., Deseo M.A., Itsiopoulos C., Jamil D., Hadi N.R., Thomas C.J. (2021). Twelve-week mediterranean diet intervention increases citrus bioflavonoid levels and reduces inflammation in people with type 2 diabetes mellitus. Nutrients.

[121] Chen S., Jiang H., Wu X., Fang J. (2016). Therapeutic effects of quercetin on inflammation, obesity, and type 2 diabetes. Mediat. Inflamm..

[122] Eid H.M., Haddad P.S. (2017). The antidiabetic potential of quercetin: Underlying mechanisms. Curr. Med. Chem..

[123] Gomez-Arbelaez D., Lahera V., Oubina P., Valero-Munoz M., de Las Heras N., Rodriguez Y., Garcia R.G., Camacho P.A., Lopez-Jaramillo P. (2013). Aged garlic extract improves adiponectin levels in subjects with metabolic syndrome: A double-blind, placebo-controlled, randomized, crossover study. Mediat. Inflamm..

[124] Shang A., Cao S.Y., Xu X.Y., Gan R.Y., Tang G.Y., Corke H., Mavumengwana V., Li H.B. (2019). Bioactive compounds and biological functions of garlic (*Allium sativum* L.). Foods.

[125] Zhu B., Qi F., Wu J., Yin G., Hua J., Zhang Q., Qin L. (2019). Red yeast rice: A systematic review of the traditional uses, chemistry, pharmacology, and quality control of an important chinese folk medicine. Front. Pharmacol..

[126] Saleem S., Muhammad G., Hussain M.A., Bukhari S.N.A. (2018). A comprehensive review of phytochemical profile, bioactives for pharmaceuticals, and pharmacological attributes of Azadirachta indica. Phytother. Res..

[127] Huang D.D., Shi G., Jiang Y., Yao C., Zhu C. (2020). A review on the potential of resveratrol in prevention and therapy of diabetes and diabetic complications. Biomed. Pharmacother..

[128] Jeyaraman M.M., Al-Yousif N.S.H., Singh Mann A., Dolinsky V.W., Rabbani R., Zarychanski R., Abou-Setta A.M. (2020). Resveratrol for adults with type 2 diabetes mellitus. Cochrane Database Syst. Rev..

[129] Ozturk E., Arslan A.K.K., Yerer M.B., Bishayee A. (2017). Resveratrol and diabetes: A critical review of clinical studies. Biomed. Pharmacother..

[130] Chen S., Zhao X., Ran L., Wan J., Wang X., Qin Y., Shu F., Gao Y., Yuan L., Zhang Q. (2015). Resveratrol improves insulin resistance, glucose and lipid metabolism in patients with non-alcoholic fatty liver disease: A randomized controlled trial. Dig. Liver Dis..

[131] Petroni M.L., Brodosi L., Marchignoli F., Musio A., Marchesini G. (2019). Moderate alcohol intake in non-alcoholic fatty liver disease: To drink or not to drink?. Nutrients.

[132] Plows J.F., Reynolds C.M., Vickers M.H., Baker P.N., Stanley J.L. (2019). Nutritional supplementation for the prevention and/or treatment of gestational diabetes mellitus. Curr. Diabetes Rep..

[133] Taylor B.L., Woodfall G.E., Sheedy K.E., O’Riley M.L., Rainbow K.A., Bramwell E.L., Kellow N.J. (2017). Effect of probiotics on metabolic outcomes in pregnant women with gestational diabetes: A systematic review and meta-analysis of randomized controlled trials. Nutrients.

[134] Bock P.M., Telo G.H., Ramalho R., Sbaraini M., Leivas G., Martins A.F., Schaan B.D. (2021). The effect of probiotics, prebiotics or synbiotics on metabolic outcomes in individuals with diabetes: A systematic review and meta-analysis. Diabetologia.

[135] Aoun A., Darwish F., Hamod N. (2020). The influence of the gut microbiome on obesity in adults and the role of probiotics, prebiotics, and synbiotics for weight loss. Prev. Nutr. Food Sci..

[136] Greger M. (2020). A whole food plant-based diet is effective for weight loss: The evidence. Am. J. Lifestyle Med..

[137] Wang L., Yang H., Huang H., Zhang C., Zuo H.X., Xu P., Niu Y.M., Wu S.S. (2019). Inulin-type fructans supplementation improves glycemic control for the prediabetes and type 2 diabetes populations: Results from a GRADE-assessed systematic review and dose-response meta-analysis of 33 randomized controlled trials. J. Transl. Med..

[138] Gao C., Rao M., Huang W., Wan Q., Yan P., Long Y., Guo M., Xu Y., Xu Y. (2019). Resistant starch ameliorated insulin resistant in patients of type 2 diabetes with obesity: A systematic review and meta-analysis. Lipids Health Dis..

[139] Rao M., Gao C., Xu L., Jiang L., Zhu J., Chen G., Law B.Y.K., Xu Y. (2019). Effect of inulin-type carbohydrates on insulin resistance in patients with type 2 diabetes and obesity: A systematic review and meta-analysis. J. Diabetes Res..

[140] Kaur B., Henry J. (2014). Micronutrient status in type 2 diabetes: A review. Adv. Food Nutr. Res..

[141] Kazi T.G., Afridi H.I., Kazi N., Jamali M.K., Arain M.B., Jalbani N., Kandhro G.A. (2008). Copper, chromium, manganese, iron, nickel, and zinc levels in biological samples of diabetes mellitus patients. Biol. Trace Elem. Res..

[142] Overbeck S., Rink L., Haase H. (2008). Modulating the immune response by oral zinc supplementation: A single approach for multiple diseases. Arch. Immunol. Ther. Exp..

[143] Jansen J., Karges W., Rink L. (2009). Zinc and diabetes—Clinical links and molecular mechanisms. J. Nutr. Biochem..

[144] Ruz M., Carrasco F., Rojas P., Basfi-Fer K., Hernandez M.C., Perez A. (2019). Nutritional effects of zinc on metabolic syndrome and type 2 diabetes: Mechanisms and main findings in human studies. Biol. Trace Elem. Res..

[145] Jayawardena R., Ranasinghe P., Galappatthy P., Malkanthi R., Constantine G., Katulanda P. (2012). Effects of zinc supplementation on diabetes mellitus: A systematic review and meta-analysis. Diabetol Metab. Syndr..

[146] Anderson R.A., Roussel A.M., Zouari N., Mahjoub S., Matheau J.M., Kerkeni A. (2001). Potential antioxidant effects of zinc and chromium supplementation in people with type 2 diabetes mellitus. J. Am. Coll. Nutr..

[147] Farvid M.S., Jalali M., Siassi F., Hosseini M. (2005). Comparison of the effects of vitamins and/or mineral supplementation on glomerular and tubular dysfunction in type 2 diabetes. Diabetes Care.

[148] Parham M., Amini M., Aminorroaya A., Heidarian E. (2008). Effect of zinc supplementation on microalbuminuria in patients with type 2 diabetes: A double blind, randomized, placebo-controlled, cross-over trial. Rev. Diabet. Stud..

[149] Roussel A.M., Kerkeni A., Zouari N., Mahjoub S., Matheau J.M., Anderson R.A. (2003). Antioxidant effects of zinc supplementation in Tunisians with type 2 diabetes mellitus. J. Am. Coll. Nutr..

[150] Marchesini G., Bugianesi E., Ronchi M., Flamia R., Thomaseth K., Pacini G. (1998). Zinc supplementation improves glucose disposal in patients with cirrhosis. Metabolism.

[151] Marchesini G., Fabbri A., Bianchi G., Brizi M., Zoli M. (1996). Zinc supplementation and amino acid-nitrogen metabolism in patients with advanced cirrhosis. Hepatology.

[152] Morgan M.Y., Blei A., Grungreiff K., Jalan R., Kircheis G., Marchesini G., Riggio O., Weissenborn K. (2007). The treatment of hepatic encephalopathy. Metab. Brain Dis..

[153] Huang H.Y., Caballero B., Chang S., Alberg A.J., Semba R.D., Schneyer C.R., Wilson R.F., Cheng T.Y., Vassy J., Prokopowicz G. (2006). The efficacy and safety of multivitamin and mineral supplement use to prevent cancer and chronic disease in adults: A systematic review for a National Institutes of Health state-of-the-science conference. Ann. Intern. Med..

[154] Chen S., Jin X., Shan Z., Li S., Yin J., Sun T., Luo C., Yang W., Yao P., Yu K. (2017). Inverse association of plasma chromium levels with newly diagnosed type 2 diabetes: A case-control study. Nutrients.

[155] Basaki M., Saeb M., Nazifi S., Shamsaei H.A. (2012). Zinc, copper, iron, and chromium concentrations in young patients with type 2 diabetes mellitus. Biol. Trace. Elem. Res..

[156] Suksomboon N., Poolsup N., Yuwanakorn A. (2014). Systematic review and meta-analysis of the efficacy and safety of chromium supplementation in diabetes. J. Clin. Pharm. Ther..

[157] Yin R.V., Phung O.J. (2015). Effect of chromium supplementation on glycated hemoglobin and fasting plasma glucose in patients with diabetes mellitus. Nutr. J..

[158] Costello R.B., Dwyer J.T., Bailey R.L. (2016). Chromium supplements for glycemic control in type 2 diabetes: Limited evidence of effectiveness. Nutr. Rev..

[159] Huang H., Chen G., Dong Y., Zhu Y., Chen H. (2018). Chromium supplementation for adjuvant treatment of type 2 diabetes mellitus: Results from a pooled analysis. Mol. Nutr. Food Res..

[160] De Valk H.W., Verkaaik R., van Rijn H.J., Geerdink R.A., Struyvenberg A. (1998). Oral magnesium supplementation in insulin-requiring Type 2 diabetic patients. Diabet. Med..

[161] Kostov K. (2019). Effects of magnesium deficiency on mechanisms of insulin resistance in type 2 diabetes: Focusing on the processes of insulin secretion and signaling. Int. J. Mol. Sci..

[162] Song Y., Manson J.E., Buring J.E., Liu S. (2004). Dietary magnesium intake in relation to plasma insulin levels and risk of type 2 diabetes in women. Diabetes Care.

[163] Guerrero-Romero F., Rascon-Pacheco R.A., Rodriguez-Moran M., de la Pena J.E., Wacher N. (2008). Hypomagnesaemia and risk for metabolic glucose disorders: A 10-year follow-up study. Eur. J. Clin. Investig..

[164] Rodriguez-Moran M., Guerrero-Romero F. (2003). Oral magnesium supplementation improves insulin sensitivity and metabolic control in type 2 diabetic subjects: A randomized double-blind controlled trial. Diabetes Care.

[165] Mooren F.C., Kruger K., Volker K., Golf S.W., Wadepuhl M., Kraus A. (2011). Oral magnesium supplementation reduces insulin resistance in non-diabetic subjects—A double-blind, placebo-controlled, randomized trial. Diabetes Obes. Metab..

[166] El Derawi W.A., Naser I.A., Taleb M.H., Abutair A.S. (2018). The effects of oral magnesium supplementation on glycemic response among type 2 diabetes patients. Nutrients.

[167] Fang X., Wang K., Han D., He X., Wei J., Zhao L., Imam M.U., Ping Z., Li Y., Xu Y. (2016). Dietary magnesium intake and the risk of cardiovascular disease, type 2 diabetes, and all-cause mortality: A dose-response meta-analysis of prospective cohort studies. BMC Med..

[168] Verma H., Garg R. (2017). Effect of magnesium supplementation on type 2 diabetes associated cardiovascular risk factors: A systematic review and meta-analysis. J. Hum. Nutr. Diet..

[169] Hannon B.A., Fairfield W.D., Adams B., Kyle T., Crow M., Thomas D.M. (2020). Use and abuse of dietary supplements in persons with diabetes. Nutr. Diabetes.

[170] Low S., Pek S., Moh A., Khin C.Y.A., Lim C.L., Ang S.F., Wang J., Ang K., Tang W.E., Lim Z. (2021). Low muscle mass is associated with progression of chronic kidney disease and albuminuria—An 8-year longitudinal study in Asians with Type 2 Diabetes. Diabetes Res. Clin. Pract..

[171] Ross E., Wright H., Villani A. (2021). Lower body extremity function is associated with health-related quality of life: A cross-sectional analysis of overweight and obese older adults with and without type 2 diabetes mellitus. Qual. Life Res..

[172] Fragala M.S., Cadore E.L., Dorgo S., Izquierdo M., Kraemer W.J., Peterson M.D., Ryan E.D. (2019). Resistance training for older adults: Position statement from the National Strength and Conditioning Association. J. Strength Cond. Res..

[173] Petroni M.L., Caletti M.T., Dalle Grave R., Bazzocchi A., Aparisi Gomez M.P., Marchesini G. (2019). Prevention and treatment of sarcopenic obesity in women. Nutrients.

[174] Chapman I., Oberoi A., Giezenaar C., Soenen S. (2021). Rational use of protein supplements in the elderly-Relevance of gastrointestinal mechanisms. Nutrients.

[175] Li Q., Wen F., Wang Y., Li S., Lin S., Qi C., Chen Z., Qiu X., Zhang Y., Zhang S. (2021). Diabetic kidney disease benefits from intensive low-protein diet: Updated systematic review and meta-analysis. Diabetes Ther..

[176] Tu D.Y., Kao F.M., Tsai S.T., Tung T.H. (2021). Sarcopenia among the elderly population: A systematic review and meta-analysis of randomized controlled trials. Healthcare.

[177] Osuka Y., Kojima N., Sasai H., Wakaba K., Miyauchi D., Tanaka K., Kim H. (2021). Effects of exercise and/or beta-hydroxy-beta-methylbutyrate supplementation on muscle mass, muscle strength, and physical performance in older women with low muscle mass: A randomized, double-blind, placebo-controlled trial. Am. J. Clin. Nutr..

[178] Manders R.J., Little J.P., Forbes S.C., Candow D.G. (2012). Insulinotropic and muscle protein synthetic effects of branched-chain amino acids: Potential therapy for type 2 diabetes and sarcopenia. Nutrients.

[179] Hey P., Gow P., Testro A.G., Apostolov R., Chapman B., Sinclair M. (2021). Nutraceuticals for the treatment of sarcopenia in chronic liver disease. Clin. Nutr. ESPEN.

[180] Kim Y. (2021). Emerging treatment options for sarcopenia in chronic liver disease. Life.

[181] Beaudart C., Buckinx F., Rabenda V., Gillain S., Cavalier E., Slomian J., Petermans J., Reginster J.Y., Bruyere O. (2014). The effects of vitamin D on skeletal muscle strength, muscle mass, and muscle power: A systematic review and meta-analysis of randomized controlled trials. J. Clin. Endocrinol. Metab..

[182] Bjelakovic G., Nikolova D., Bjelakovic M., Gluud C. (2017). Vitamin D supplementation for chronic liver diseases in adults. Cochrane Database Syst. Rev..

[183] Braganca A.C., Alvares-da-Silva M.R. (2010). Prevalence of diabetes mellitus and impaired glucose tolerance in patients with decompensated cirrhosis being evaluated for liver transplantation: The utility of oral glucose tolerance test. Arq. Gastroenterol..

[184] Elkrief L., Rautou P.E., Sarin S., Valla D., Paradis V., Moreau R. (2016). Diabetes mellitus in patients with cirrhosis: Clinical implications and management. Liver Int..

[185] Kallwitz E.R. (2015). Sarcopenia and liver transplant: The relevance of too little muscle mass. World J. Gastroenterol..

[186] Schiavo L., Busetto L., Cesaretti M., Zelber-Sagi S., Deutsch L., Iannelli A. (2018). Nutritional issues in patients with obesity and cirrhosis. World J. Gastroenterol..

[187] Brodosi L., Petta S., Petroni M.L., Marchesini G., Morelli M.C. (2021). Management of diabetes in candidates for liver transplantation and in transplant recipients. Transplantation.

[188] Colosimo S., Ravaioli F., Petroni M.L., Brodosi L., Marchignoli F., Barbanti F.A., Sasdelli A.S., Marchesini G., Pironi L. (2021). Effects of antidiabetic agents on steatosis and fibrosis biomarkers in type 2 diabetes: A real-world data analysis. Liver Int..

[189] Vukotic R., Raimondi F., Brodosi L., Vitale G., Petroni M.L., Marchesini G., Andreone P. (2020). The effect of liraglutide on beta-blockade for preventing variceal bleeding: A case series. Ann. Intern. Med..

[190] Bischoff S.C., Bernal W., Dasarathy S., Merli M., Plank L.D., Schutz T., Plauth M. (2020). ESPEN practical guideline: Clinical nutrition in liver disease. Clin. Nutr..

[191] Plank L.D., Gane E.J., Peng S., Muthu C., Mathur S., Gillanders L., McIlroy K., Donaghy A.J., McCall J.L. (2008). Nocturnal nutritional supplementation improves total body protein status of patients with liver cirrhosis: A randomized 12-month trial. Hepatology.

[192] Verboeket-van de Venne W.P., Westerterp K.R., van Hoek B., Swart G.R. (1995). Energy expenditure and substrate metabolism in patients with cirrhosis of the liver: Effects of the pattern of food intake. Gut.

[193] Baldassarre M., Naldi M., Zaccherini G., Bartoletti M., Antognoli A., Laggetta M., Gagliardi M., Tufoni M., Domenicali M., Waterstradt K. (2021). Determination of effective albumin in patients with decompensated cirrhosis: Clinical and prognostic implications. Hepatology.

[194] European Association for the Study of the Liver (2018). EASL Clinical Practice Guidelines for the management of patients with decompensated cirrhosis. J. Hepatol..

[195] Caraceni P., Pavesi M., Baldassarre M., Bernardi M., Arroyo V. (2018). The use of human albumin in patients with cirrhosis: A European survey. Expert Rev. Gastroenterol. Hepatol..

[196] Caraceni P., Riggio O., Angeli P., Alessandria C., Neri S., Foschi F.G., Levantesi F., Airoldi A., Boccia S., Svegliati-Baroni G. (2018). Long-term albumin administration in decompensated cirrhosis (ANSWER): An open-label randomised trial. Lancet.

[197] Goldstein-Fuchs J., Kalantar-Zadeh K. (2015). Nutrition Intervention for advanced stages of diabetic kiidney disease. Diabetes Spectr..

[198] Syauqy A., Hsu C.Y., Lee H.A., Rau H.H., Chao J.C. (2020). Association between dietary patterns and kidney function parameters in adults with metabolic syndrome: A cross-sectional study. Nutrients.

[199] Ko G.J., Kalantar-Zadeh K., Goldstein-Fuchs J., Rhee C.M. (2017). Dietary approaches in the management of diabetic patients with kidney disease. Nutrients.

[200] Isaka Y. (2021). Optimal protein intake in pre-dialysis chronic kidney disease patients with sarcopenia: An overview. Nutrients.

[201] Kidney Disease Ourcomes Quality Initiative (2007). KDOQI clinical practice guidelines and clinical practice recommendations for diabetes and chronic kidney disease. Am. J. Kidney Dis..

[202] Kalantar-Zadeh K., Joshi S., Schlueter R., Cooke J., Brown-Tortorici A., Donnelly M., Schulman S., Lau W.L., Rhee C.M., Streja E. (2020). Plant-dominant low-protein diet for conservative management of chronic kidney disease. Nutrients.

[203] Tuttle K.R., Bakris G.L., Bilous R.W., Chiang J.L., de Boer I.H., Goldstein-Fuchs J., Hirsch I.B., Kalantar-Zadeh K., Narva A.S., Navaneethan S.D. (2014). Diabetic kidney disease: A report from an ADA Consensus Conference. Am. J. Kidney Dis..

[204] Shapiro H., Theilla M., Attal-Singer J., Singer P. (2011). Effects of polyunsaturated fatty acid consumption in diabetic nephropathy. Nat. Rev. Nephrol..

[205] Kong Y.W., Baqar S., Jerums G., Ekinci E.I. (2016). Sodium and its role in cardiovascular disease—The debate continues. Front. Endocrinol..

[206] Abe M., Hamano T., Hoshino J., Wada A., Inaba M., Nakai S., Masakane I. (2017). Is there a “burnt-out diabetes” phenomenon in patients on hemodialysis?. Diabetes Res. Clin. Pract..

